# Single-fiber three-dimensional shape sensing via femtosecond laser inscribed orthogonal eccentric scatterers

**DOI:** 10.1038/s41377-026-02425-z

**Published:** 2026-08-10

**Authors:** Pengtao Luo, Fengyi Chen, Tuan Guo, Xueguang Qiao, Ruohui Wang

**Affiliations:** 1 Xi’an Key Laboratory of Optical Fiber Sensing Technology for Underground Resources, School of Physics, Northwest University, Xi’an, China; 2 Institute of Photonics Technology, College of Physics & Optoelectronic Engineering, Jinan University, Guangzhou, China

**Keywords:** Optical sensors, Fibre optics and optical communications

## Abstract

Optical fiber sensors have emerged as a powerful platform for high-precision shape detection in soft robotics and minimally invasive medical devices. However, existing fiber-sensing architectures face a long-standing trade-off between device size and spatial resolution. This work reports a miniaturized three-dimensional shape-sensing system based on misplaced orthogonal eccentric scatterers (MOESs) densely inscribed into the cladding of a standard single-mode fiber. The MOESs array is fabricated through a scalable reel-to-reel femtosecond laser writing process. Each MOES element functions as a curvature-dependent Rayleigh scatterer, and their misplaced orthogonal arrangement spatially encodes full 3D deformation information within a single fiber core. Experimental results demonstrate high-fidelity 2D bending and full 3D deformation reconstruction. The intensity-domain reconstruction algorithm further exhibits strong immunity to environmental perturbations. This approach defines a new paradigm for compact, cost-effective, and high-fidelity fiber sensors, paving the way for applications in unstructured and dynamic environments.

## Abstract (web summary)

We propose a novel 3D shape-sensing mechanism with misplaced orthogonal eccentric scatterers arrays in SMF. This engineered scatterers enables curvature-dependent Rayleigh intensity modulation, allowing 3D shape data to be encoded.

## Graphical abstract

**Figure:**
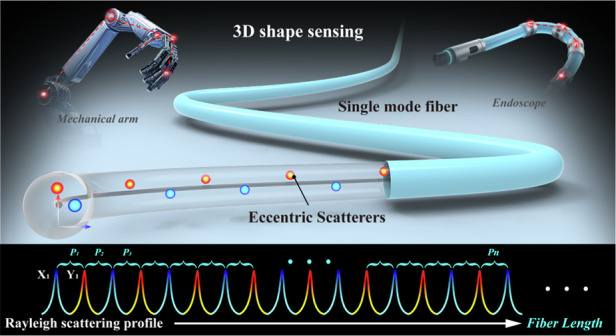

## Introduction

Soft robotics has gained significant traction globally, with its superior compliance, flexibility, and robustness widely recognized^1,2^. These attributes have enabled breakthroughs in various fields, such as in-pipe inspection^3^, drug delivery^4,5^, and minimally invasive surgery^6^. As applications expand, the demand for higher integration^7,8^ and simplification^9^ of core components-actuation, sensing, and control-has intensified. Among these, sensing systems are indispensable for providing navigation foundations for actuation and control systems^10^. Soft robotic systems increasingly require high-precision, high-resolution spatial position and posture monitoring, especially for complex tasks such as proprioceptive^11,12^ and full-state feedback control^13^. Currently, electrically-based sensors^14,15^ (e.g., resistive and capacitive sensors) remain the mainstream choice, as they are more accessible and compatible with electrically driven and controlled robotic systems. However, these sensors face notable challenges in repeatability and large-scale integration, limiting their further adoption in soft robotics^16^.

With the advancement of interdisciplinary technologies, optoelectronic sensing has emerged as a promising alternative to address these limitations^17^. Fiber-optic sensing technology, particularly three-dimensional deformation encoding, has opened new avenues in soft robotics^18^. Fiber-optic deformation sensors enable real-time measurement of spatial information, including orientation, trajectory, and position of the fiber or attached objects^19,20^. Compared to traditional electrical sensors, fiber-optic sensors offer advantages such as compact size, flexibility, high precision, immunity to electromagnetic interference, and in situ monitoring capabilities^21^. This significantly enhances the applicability of soft robots in complex environments, including precise motion control, state feedback, and environmental adaptability^22^.

Fiber-optic shape sensing integrates single-point bending sensing structures with distributed or quasi-distributed fiber sensing techniques to enable continuous or quasi-continuous deformation reconstruction^23^. While numerous fiber bending sensors, such as Mach-Zehnder interferometers (MZIs)^24^ and Fabry-Pérot interferometers^25^, have been developed, only distributed optical frequency domain reflectometry (OFDR)^26,27^ and quasi-distributed fiber Bragg gratings (FBGs)^28–31^ have approached truly continuous deformation measurement. These typical OFDR and FBG techniques often require multi-channel fiber bundles or multi-core fibers as carriers. Commercial products of fiber-optic sensors based on basic principles have emerged for the medical and robotics applicaitons^32,33^. Yet, as future scenarios demand higher precision, lower energy consumption, and seamless integration, current technologies face two major challenges. First, the sensor’s size must be further reduced by abandoning bulky multi-fiber or multi-core designs in favor of compact single-fiber configurations, thereby optimizing space utilization in soft robotics. Second, the demodulation system requires simplified implementation through single-channel signal processing, enabling richer deformation information extraction with minimal data input while reducing costs and accelerating signal processing.

Several schemes have been proposed to address these challenges. Optical switching can sequentially interrogate multiple channels, but this inevitably compromises speed and real-time feedback. Rayleigh-signature domain multiplexing offers an alternative by using cross-correlation to demix multi-channel scattering signals, yet its robustness under noisy and dynamic environments remains uncertain^34^. These limitations underscore the urgent need for new sensing schemes that can simultaneously deliver high spatial resolution, reliable feature recognition, and compact architectures.

Recent advancements in femtosecond laser direct writing technology have facilitated the development of specialized devices, such as cladding FBGs^35^, off-axis FBGs^36^, and off-axis MZIs^37^. These structures enable single-point one-dimensional or two-dimensional bending measurement within a single fiber. However, for deformation measurement, continuous two-dimensional bending measurements are essential for reconstructing curves. Efforts have been made to explore single-channel FBGs^38–40^, leveraging their wavelength-marked features for integrated bending and deformation encoding. Yet, this wavelength-based identification also poses limitations: the cumulative spectral bandwidth of FBGs must remain within the bandwidth of the light source. This wavelength constraint becomes increasingly prominent in single-channel sensing, where current studies report fewer than ten gratings per channel and unsatisfactory spectral quality^38,40^. From the perspective of sensing, OFDR is a continuous measurement technique with near-unlimited sensing points along the sensing distance. However, because it lacks wavelength markers, single-channel OFDR cannot distinguish two-dimensional bending angles. Taken together, these observations point toward the ideal solution: a single-channel fiber-optic deformation sensor that combines the wavelength-specific feature recognition of FBGs with the dense, near-unlimited spatial sampling of OFDR.

In this study, we present a novel fiber-optic deformation encoding technology for shape sensing based on a misplaced orthogonal eccentric scatterers (MOESs) array. These eccentric scatterers are distributed within the cladding of a single-mode fiber, overlapping with the edges of the electric field mode. Unlike conventional FBG- or OFDR-based approaches that rely on spectral multiplexing to encode deformation information, the proposed scheme employs a spatial encoding strategy, in which three-dimensional deformation is mapped to the deliberately designed spatial misplacement of orthogonal eccentric scatterers. Using OFDR technology, we are able to detect an enhanced scattering intensity at characteristic points, where the backward signal intensity is correlated with fiber curvature. Here, OFDR serves as a single-channel spatial readout tool, while the deformation encoding itself is achieved through the spatial configuration of the scatterers rather than wavelength-domain discrimination. The eccentric scatterers array is designed with a misplaced orthogonal arrangement along the fiber, enabling the extraction of 3D deformation information from a single OFDR channel. We have developed a reel-to-reel femtosecond laser direct writing technique to fabricate the MOESs array in the sensing fiber. The ability of the orthogonally distributed eccentric scatterers to measure 2D bending at a single point has been validated. Further, we demonstrate the reconstruction of 3D deformation using a sensing fiber containing 36 eccentric scatterers. Finally, we measured the anti-environmental interference capability of the deformation reconstruction scheme based on energy detection. The deformation encoding approach based on the MOESs array offers several advantages: (i) The sensor only uses one single-core standard SMF, eliminating the need for multi-channel demodulation equipment and achieving a near-limit size; (ii) The scatterers spectra are wavelength-independent, and the number of sensing points along the fiber length is limited only by loss; (iii) Bending information is obtained from intensity variations, providing excellent immunity to strain and temperature induced interference. Through these advancements, we aim to open up new opportunities for highly integrated and simplified sensing solutions for complex deformation requirements.

## Results

### MOESs array shape encoding scheme

**Fig. 1:**
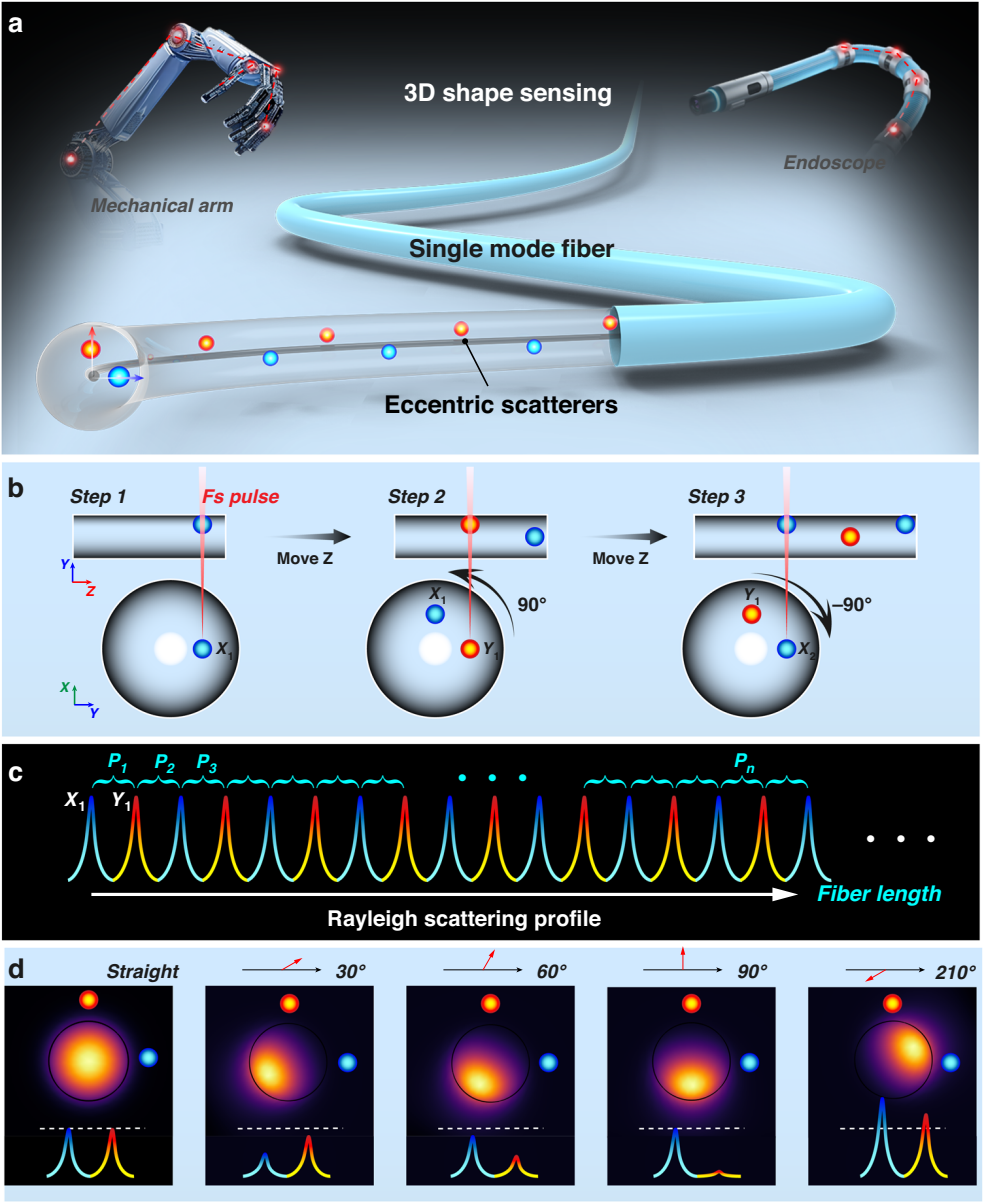
Design concept for deformation encoding using MOESs array fiber. **a** Schematic of the deformation encoding scheme based on eccentric scatterers. **b** The preparation steps for a group of MOESs. **c** The coding rule for the MOESs array, each scatterer is utilized twice. **d** Schematic diagram of the distribution of mode fields around the fiber core with bending, including the relative variation of MOESs peak intensity

The fiber deformation encoding scheme based on the MOESs array is illustrated in Fig. 1a, showing the arrangement of the eccentric scatterers within the single-mode fiber and the fiber’s deformation after bending. The rationale for selecting scatterers as the sensing elements lies in their ultra-low loss and wavelength-independent properties, enabling virtually unlimited sensor points. To realize three-dimensional deformation measurement capabilities for the scatterers, an in-depth analysis of the cladding-enhanced scattering structure^41^ was conducted. It was found that even with a single scatterer prepared at an eccentric position, a prominent scattering enhancement peak can be observed in the backscattering profile measured using OFDR. The intensity of this peak depends on the overlap between the scatterer and the distribution of the probing light field.

Figure 1b shows the preparation steps for a group of MOESs. After the preparation of the previous eccentric scatterer is completed, the fiber needs to be translated (by the misplacement distance) and rotated by 90 degrees to prepare the next scatterer. The complete preparation steps for the MOESs array are described in the “Methods”. The reel-to-reel femtosecond laser direct writing system improved for the preparation of the MOESs array is described in the Fig. S1.

Compared to off-axis FBGs^40^, the unique feature of eccentric scatterers is wavelength-independent. However, this advantage also imposes a limitation: two orthogonal off-axis FBGs at different wavelengths can achieve single-point 2D deformation measurements, while orthogonal eccentric scatterers lack distinguishing spectral features. To address this issue, the key characteristics of FBGs and eccentric scatterers were analyzed-FBGs rely on lightwave frequency, whereas eccentric scatterers depend on temporal intensity. By introducing temporal displacement in the signals of orthogonal structures, the two orthogonal signals can be separated based on their temporal sequence. OFDR technology can convert the measured time-domain signals into positional information, making it possible to distinguish orthogonal directions by fixing orthogonal scatterers at specific spatial misplacements along the fiber, through this configuration, two adjacent scatterers form one sensing point, and each scatterer is utilized twice. The coding rule is shown in Fig. 1c. When the misplacement distance exceeds the spatial resolution of the OFDR measurement, discrete scattering enhancement peaks can be generated, thereby separating useful data. Detailed information about the OFDR measurement setup is described in the *Methods*.

When the fiber bends, the electric mode field center of the probing light shifts opposite to the bending direction, leading to variations in the intensity of the reflection enhancement peak. Figure 1d shows the variation of the electric mode field near the core in several bending states and the corresponding variation trend of the scatterers peak.

Unlike typical OFDR technology, the process of acquiring MOESs signals is more simplified-only a single-step FFT needs to be performed on the interfering frequency-swept signal to obtain the Rayleigh scattering profile (Figure S2). This significantly saves the time required for signal acquisition. In other words, we only use the OFDR system as an OTDR with high spatial resolution here. This is necessary to achieve accurate scattering intensity and high spatial resolution.

**Fig. 2:**
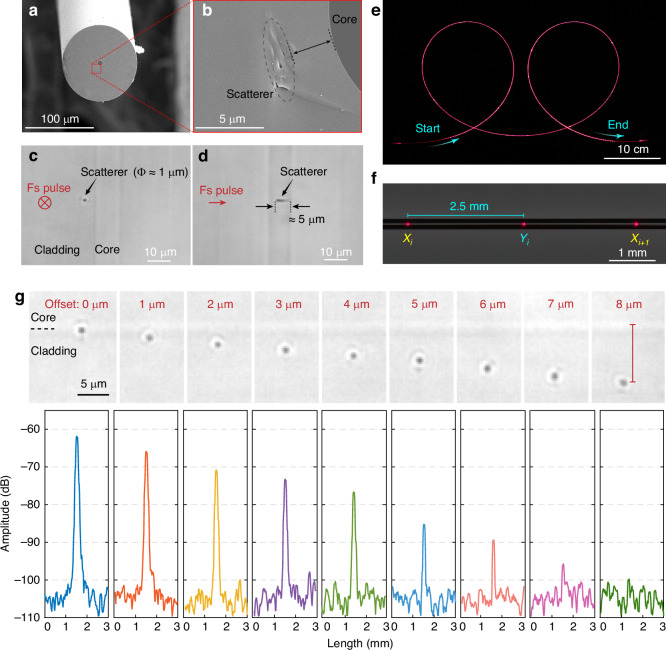
The basic properties of the eccentric scatterers. **a** SEM image of cross section of single mode fiber with scatterer. **b** SEM image of the scatterer’s morphology, showing the presence of micro-explosions and nanoscale gratings. **c**, **d** Micrograph of a refractive index modulation point generated by a femtosecond pulse at the eccentric position of the fiber, observed both perpendicular (**c**) and parallel (**d**) to the laser incident direction. **e** The intuitive scattering effect of red light on the MOESs array fiber, **f** The amplified part of red light scattering can clearly distinguish the scattering effect of each scatterer. **g** Backscattering profiles of eccentric scatterers with different offsets, showing that the backscattering signal strength aligns with the intensity distribution of the detected optical field mode

As shown in Fig. 2a and b, the morphology of the nanometer range of the modulation region is observed using SEM by truncating the modulation point cross-section. The presence of micropores in the modulated region indicates Coulomb explosions^42^, which demonstrate exceptional stability. Additionally, strip-shaped nano-grating structures are distributed around the micropores along the laser propagation direction^43,44^, with a period of hundreds of nanometers, matching the scale of Rayleigh scattering enhancement for probing light.

Figure 2c and d show the morphology of modulation points in the fiber observed using a coaxial optical focusing-microscopy setup after fabrication. The laser incidence direction is indicated, and the color differences reveal the modulation points as ellipsoidal refractive index-modulated regions along the laser incidence direction, approximately 1×1×5 µm in size.

When red light is launched into the MOESs fiber, the scattering induced by the inscribed scatterers on the transmitted light becomes clearly visible, as shown in Fig. 2e. The magnified region in Fig. 2f illustrates the macroscopic scattering behavior of individual eccentric scatterers under red-light illumination. Due to the small eccentric offset (~4 µm) relative to the fiber diameter, the microscopic transverse displacement and orthogonal configuration of adjacent scatterers are not directly resolvable in this image. Nevertheless, individual scatterers can still be clearly distinguished through their scattering responses and the precisely controlled axial spacing between adjacent scatterers.

Different eccentric positions of scatterers were fabricated, as shown in Fig. 2g. Scatterers outside the core were fabricated starting from the core-cladding interface, with a stepping distance of 1 µm for each scatterer. It is observed that with the increase of offset, the intensity of backscattered signal gradually decreases, which is basically consistent with the contour of Gaussian distribution of mode field. Based on this result, the fabrication position of the eccentric scatterers in the experiment was chosen to be less than 6 µm offset to ensure clear scattering intensity signals.

### 2D bending measurement

**Fig. 3:**
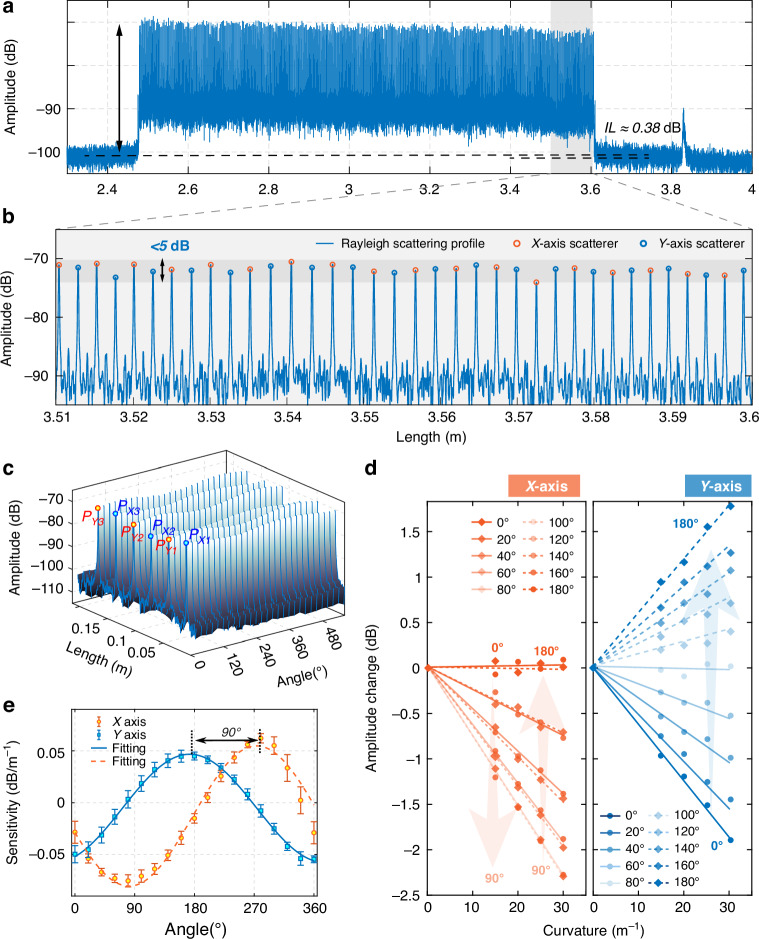
Scattering profiles of MOESs array and its 2D curvature measurement. **a** Backscattering profiles of 1.13 m MOESs fiber, with an offset of 4 µm and a misplacement of 2.5 mm. **b** Backscattering profiles of the tested fiber for demonstration purposes. The total sensor length is 9 cm, with peaks at odd positions representing X-axis scatterers and peaks at even positions representing Y-axis scatterers. **c** Scattering intensity variations for six adjacent scatterers at a curvature of 20 m^−1^, as the bending direction changes by 540°. **d** Bending response of X-axis and Y-axis scatterers, showing the trend of curvature sensitivity ***s*** as the rotation device is turned 180°, with a phase difference of approximately 90°. **e** Curvature sensitivity trends for the X and Y-axis over a 360° rotation, showing a sinusoidal variation with a phase shift of 90°, indicating 2D measurement capability

A 1.13-meter-long fiber with a MOESs array was fabricated using an improved reel-to-reel femtosecond laser direct writing method. As shown in Fig. 3a, a total of 450 scatterers were integrated, representing 449 sensing points. Each scatterer has an offset of 4 μm and a misplacement distance of 2.5 mm, enabling deformation measurement with a spatial resolution of 2.5 mm. By accurately recording the difference in the intensity of backscattered signals between the front and rear ends of the scatterers array, the MOESs fiber loss can be precisely evaluated. Experimental results show that the total loss caused by the 450 eccentric scatterers is approximately 0.38 dB. The ultra-low insertion loss indicates that this system significantly outperforms FBG sensors in terms of high-density and large-scale integration capabilities. Furthermore, owing to the low insertion loss of the MOESs and the favorable power budget of OFDR interrogation, the achievable sensing length is not fundamentally limited by optical attenuation, but is primarily by cumulative reconstruction error at extended distances.

For demonstration purposes, a sensing fiber containing 36 eccentric scatterers was selected for deformation coding testing. As shown in Fig. 3b, the backscattering profile shows that the signal intensity of each scatterer is enhanced by more than 30 dB, and the amplitude difference between scatterers is less than 5 dB. This difference stems from the positioning accuracy during the manufacturing process and fluctuations in laser pulse power. The signal peaks are classified according to their corresponding scatterer positions in the order of the X-axis and Y-axis.

The eccentric scatterers, arranged in misplaced orthogonal, were used to measure 2D bending curvatures and angles, forming the basis for 3D deformation encoding. A fiber-guiding tool with a defined reference curvature was developed. The sensing fiber was placed on a track with fixed curvature, and the bending direction was controlled using a rotary device. Reflection intensity variations were recorded under different bending curvatures and angles. Figure 3c shows the intensity variations of six adjacent scatterers when the fiber was rotated by 540° at a curvature C = 20 m^−1^. Each scatterer’s intensity follows a sinusoidal variation, with the peaks of orthogonal scatterers differing by 90°, demonstrating the capability of eccentric scatterers to identify both curvature and angle.

To investigate the response characteristics of eccentric scatterers under different curvature conditions, the peak intensities at varying curvatures were measured. Results indicate that the amplitude of intensity variations increases with curvature, further confirming the high sensitivity of eccentric scatterers to curvature changes. Figure 3d illustrate the intensity variations of scatterers along two axes during a continuous 90° rotation, showing intensity values at different curvatures. The slope represents curvature sensitivity ***s***, revealing consistent patterns of intensity variations at different bending angles. Figure 3e presents statistical data on curvature sensitivity after a 360° rotation of the fiber. Due to positioning accuracy and intensity noise, the maximum sensitivity of eccentric scatterers exhibits some variation. Through sinusoidal fitting, the trend of curvature-intensity sensitivity variation at different angles was obtained, and the average maximum sensitivity was calculated to be ~0.05 dB/m^−1^. To simplify the computation process, the sensitivity ***s*** was treated as a constant (0.05 dB/m^−1^) during curvature and angle calculations.

In practice, the measurable curvature range is primarily constrained by the mechanical bending limit of standard single-mode fibers. For a minimum bending radius of ~5 mm (corresponding to a curvature of ~200 m^-1^, standard G.652D single-mode fibers), the expected intensity variation of individual scatterers is below 10 dB, which remains well within the >30 dB scattering enhancement margin provided by the MOESs. Consequently, the enhanced scattering peaks remain clearly resolvable throughout the entire practical curvature range.

### 3D shape sensing

**Fig. 4:**
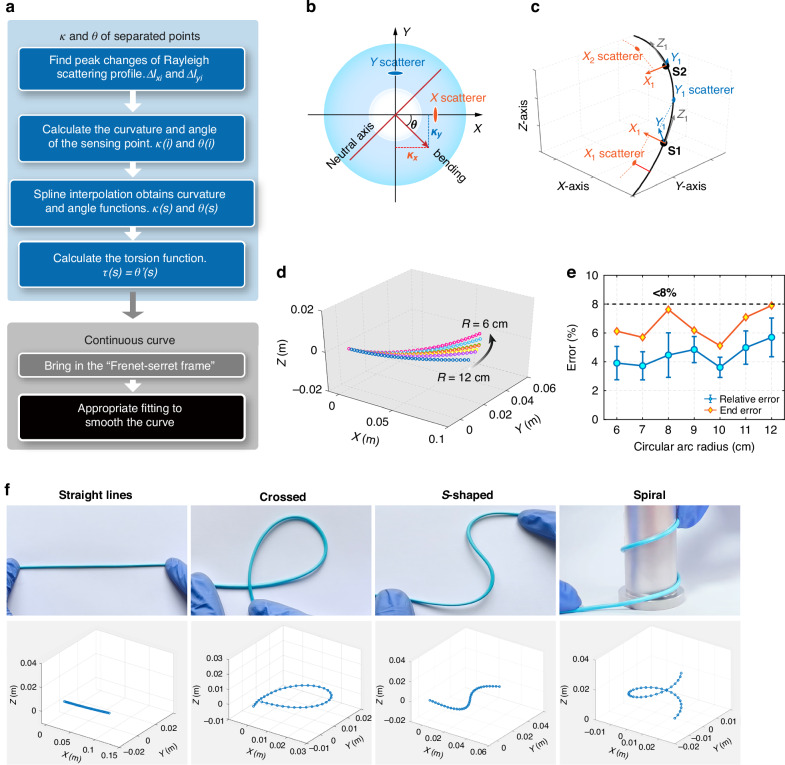
Experimental validation of 3D shape sensing. **a** Deformation reconstruction workflow based on MOESs array fiber. **b** Spatial relationship between eccentric scatterers, fiber mode fields, and bending directions in the encoding process. **c** Projection method for scatterers at sensor points, where each pair of adjacent scatterers encodes the deformation at a single sensor point. **d** Deformation reconstruction results for circular arc shapes with radii of 6, 7, 8, 9, 10, 11, and 12 cm, with a total curve length of 8.75 cm and a sensor resolution of 2.5 mm. **e** Reconstruction errors for the seven reconstructed curves compared to the standard circular arc, categorized into endpoint errors and relative errors, with all errors less than 8%. **f** Deformation reconstruction of randomly bending shapes using the MOESs array shape sensor, including straight lines, irregular bending, crossed, and S-shaped bending

After verifying that the MOESs can achieve single-point 2D bending measurement, we utilized the previously described array of eccentric scatterers to reconstruct the 3D deformation. Figure 4a outlines the reconstruction workflow based on eccentric scatterers: First, the peak intensity of each scatterer is measured while the fiber is in its straight state, and these values are taken as references. When the fiber bends, changes in the peak intensities relative to the reference values are recorded. Using the maximum curvature sensitivity ***s***, the bending curvature and angle at each sensing point can be determined. Next, separate the scatterers in two directions, and spline functions are applied to interpolate the discrete curvature and angle values, yielding the curvature and angle functions. Differentiating the angle function provides the torsion function. By incorporating the curvature and torsion functions into the Frenet-Serret framework equations and numerically solving them, the tangent vector function along the curve is obtained. Finally, integrating the tangent vector function along the arc length s reconstructs the curve’s spatial coordinates^45^.

The method for calculating 2D bending based on calibrated curvature sensitivity is as follows: with a known offset distance, the product of intensity variation and maximum sensitivity represents the projection in that direction. Using the projection length and offset distance, the angle between the selected point and the direction of maximum ***s*** can be calculated. The bending direction relative to the reference coordinate system can be determined using orthogonal scatterer positions. Bending curvature can then be calculated based on the right-angled triangle relationship using the projection intensities in two orthogonal directions. Based on the positional relationships shown in Fig. 4b and the previously described bending calculation methods, bending information can be extracted by monitoring changes in the scatterers.

Figure 4c illustrates the relationship between sensing points and eccentric scatterers. The midpoint between two adjacent scatterers can be considered as a sensing point; in this case, the adjacent scatterers can act as the X and Y-axis measurement units for each sensing point. This configuration allows for 35 sensing points to be measured for bending using the 36 eccentric scatterers. The distance between each scatterer and its corresponding sensing point is only 1.25 mm, allowing the scatterer’s bending to be approximately considered as the bending along a single axis at the sensing point.

The deformation encoding validation was conducted by arranging the sensing fiber into circular arcs of different radii: 6, 7, 8, 9, 10, 11, and 12 cm. The curvature, angle, and torsion functions derived from the measured intensity changes are shown in Figure S6. The stability of the measurements was primarily limited by fluctuations in intensity measurements. Deformation encoding results based on the Frenet-Serret framework using the data are presented in Fig. 4d. To achieve smoother curves, the curvature and torsion functions were pre-smoothed. Two evaluation metrics-end error and relative error-were used to assess reconstruction accuracy^28^, as defined in “Methods”. The calculated errors, plotted in Fig. 4e, indicate that the end error and relative error were below 8% and 6%, respectively. The higher end error compared to the relative error is attributed to cumulative errors introduced during integration within the Frenet-Serret framework. The cumulative error leads to a gradual increase in the end error, and its variation trend is shown in Fig. S7.

Figure 4f presents reconstruction results for randomly bent shapes, including crossed, S-shaped, and spiral bending. For clear visualization of the fiber shape against the background, the MOESs fiber was loosely encapsulated in a blue PVC tube (outer diameter: 1 mm, inner diameter: 0.5 mm), and the reconstructed shape was compared with its actual configuration. Although the reconstruction error for these random shapes was not quantitatively evaluated, it is visually evident that various bending postures can be reliably identified.

### Anti-environmental interference capability

Energy distribution in SMFs is intrinsically associated with their loss mechanisms. While SMFs exhibit strong resistance to loss under normal conditions, fiber deformation caused by external environmental factors-such as temperature and strain-can significantly alter bending losses. Temperature variations affect the refractive index of the fiber material, influencing mode coupling between the core and cladding^46^. Strain-induced stress distribution causes asymmetric displacements, disrupting the weakly guiding state of the fiber due to the elastic-optic effect, which may lead to inaccuracies in energy measurements under varying bending stresses^40^. These alterations complicate deformation encoding based on reflection intensity. Existing models describe bending loss mechanisms^47^; this study further investigates the interplay between environmental and shape encoding.

**Fig. 5:**
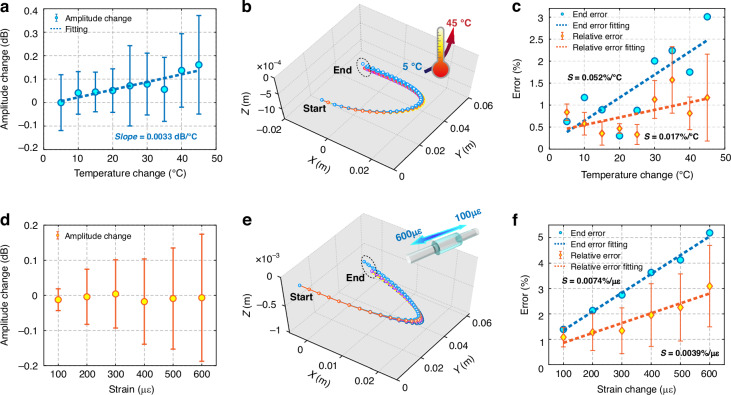
Influence of environmental interference on deformation encoding. Temperature change: **a** Corresponding changes in peak scattering intensity. **b** Reconstructed deformation. **c** End errors and relative errors as functions of temperature. Strain change: **d** Corresponding changes in peak scattering intensity. **e** Reconstructed deformation. **f** End errors and relative errors as functions of strain

Figure S8 shows the variation of backscattering profile, and the relationship between temperature and scattering signal amplitude as the temperature increases from 20°C to 60°C is shown in Fig. 5a. The scattering intensity of MOESs array fiber shows excellent thermal stability. Using this result, reconstructed deformations of the fiber under fixed conditions at different temperatures are shown in Fig. 5b, with corresponding error statistics summarized in Fig. 5c. The end error and relative error varied at rates of 0.052%/°C and 0.017%/°C, respectively.

Figures 5d and S9 illustrate the MOESs fiber’s response to strain. As strain increased, measurement errors in peak signal amplitude became more pronounced (Fig. 5d). However, averaging across multiple measurements yielded stable results. The variations in end error and relative error were 0.0074%/µε and 0.0039%/µε, respectively.

The shape sensing technique of MOESs arrays was compared with other methods, by means of five indicators: number of sensing points, size (sensor diameter), measurement error, number of channels and the interference resistance. As shown in Fig. S10, the MOESs array fiber not only features a single channel and small size but also has a far greater number of sensing points than other methods. From the perspective of measurement accuracy, the eccentric scatterers approach achieves results comparable to those of OFDR and FBG technologies. Notably, in addition to advantages in spatial resolution and system simplicity, the eccentric scatterer-based deformation sensor only requires the sensing region to be within the mode field diameter of a single-mode fiber. This suggests the feasibility of fabricating even more compact deformation sensors using fibers with smaller cladding diameters down to 80 or 50 µm.

## Discussion

This study presents, for the first time, a novel 3D fiber shape sensor with single-core standard single-mode fiber. Through the innovative design of misplaced orthogonal sensor configurations, 3D deformation encoding with high spatial resolution and no wavelength limitations was achieved using one-dimensional OFDR detection signals. To fabricate the sensor, we developed a scalable reel-to-reel femtosecond laser direct writing system with capabilities for fiber rotation and long-distance translation, enabling the precise fabrication of MOESs array fiber. The sensor’s accuracy rivals that of traditional methods while offering notable advantages, including reduced size and enhanced spatial resolution. Finally, we validated that the energy-based detection scheme exhibits minimal sensitivity to temperature and strain, underscoring its robustness. These findings demonstrate the potential of MOESs deformation encoding sensors to provide reliable trajectory guidance in confined spaces and harsh environments.

The primary limitation to further improving reconstruction accuracy lies in fluctuations in the detection of weak scattered signals. The reflectivity of the eccentric scatterers, approximately -70 dB, imposes stringent requirements on the stability of the light source and the noise performance of the detection system. Beyond upgrading the experimental setup, future work will focus on leveraging deep learning to mitigate the impact of energy fluctuations^48,49^. Deep learning techniques, such as autoencoders and generative adversarial networks, could significantly enhance the system’s ability to extract meaningful features from noisy and complex signals while reducing the effects of other interferences. In practical applications, the fiber may experience sharp bending with small radii, leading to bending-induced loss and affecting the amplitude of subsequent MOESs signals. To address for this effect, we introduce the concept of on-axis reference scatterers as a potential approach for distinguishing such conditions, as illustrated in Fig. S12.

Despite certain limitations, this work has showcased the distinctive advantages and vast potential of eccentric scatterers as an innovative deformation encoding structure. Overall, the eccentric scatterers sensor overcomes the inherent trade-offs between size and spatial resolution faced by traditional deformation encoding sensors. Remarkably, even in fibers with diameters smaller than 100 µm and detection systems capable of achieving sub-millimeter spatial resolution, eccentric scatterers remain applicable. This positions it as an advanced deformation encoding technology capable of pushing the boundaries of size and resolution limits. Its compatibility lends itself to broad application potential across diverse fields, including advanced artificial perception systems, high-precision vibration detection, robotic shape sensing, and other frontier technologies. Notably, the development of miniaturized sensors could further accelerate progress in prototype design, enabling enhanced perception capabilities and more seamless system integration.

## Methods

### Sensor fabrication

The improved reel-to-reel femtosecond laser direct writing system is shown in Figure S1. The fiber movement control system consists of a fiber spool, cleaning device, tension controller, and limit pulleys. The tension in the fiber is set to 40 g by the tension controller, ensuring stable tension and enhancing the consistency of eccentric scatterers fabrication. The femtosecond laser used (ULTRON Photonics Inc.) has a central wavelength of 515 nm and a pulse width of 300 fs, with single-pulse energy set at 0.5 µJ. After shaping, the femtosecond laser beam is circularly polarized with a spot diameter of approximately 6 mm. A plane light source with a wavelength of approximately 650 nm is used for transmission illumination, and a dichroic mirror separates the femtosecond laser from the illumination light. The illumination light is further shaped to eliminate chromatic aberrations from the lens and objective, ensuring that the focal plane and field of view coincide. A 100× oil immersion objective (NA = 1.25) is used for focusing. The specially designed fiber rotation fixture is fixed on a three-axis air-cushion displacement stage (AUSPRECISION, QFL100-100XY-5Z) to allow precise adjustment of the fiber position.

The fabrication process of the MOESs array is as follows: After shaping the optical path, the femtosecond laser focus is adjusted to the center of the CCD camera’s field of view. The fiber is then connected to the spool, and the rotation fixture is used to fix the fiber. The fiber is adjusted with the aid of the synchronized observation camera, ensuring the laser focus is directed at the eccentric position. When the laser is activated, the femtosecond laser interacts with the fiber, forming scatterers. The fiber is moved axially by 2.5 mm using the air-cushion displacement stage, then rotated 90° by the rotation fixture. The relative positions of the fiber and laser focus are adjusted again, completing one set of MOESs. The rotation fixture is then reversed 90° to return the fiber to its initial orientation. The preparation of such one set of MOESs takes about 30 s. The rotation fixture is released, and the fiber is moved to the next position by the spool, repeating the process for subsequent scatterers. A video showing the fabrication process of a set of scatterers is provided as *Video 1*. The final sensor fiber is completed.

### OFDR measurement setup

The OFDR measurement setup includes a light source with a scanning width of 42 nm and a center wavelength of 1566 nm. The spatial resolution is 100 µm. Here, OFDR serves only to localize and quantify the backscattering intensity from the engineered scatterers, while the 3D deformation information is encoded in the spatial misplacement of the scatterers rather than in wavelength-domain features.

### Shape reconstruction method

The detailed procedure for shape reconstruction is outlined as follows.:

Step 1: The reflected peak intensities of the MOESs array are first recorded in the straight state as a reference. The difference between the reference and the intensities measured during bending yields the energy change, Δ*I*_*i*_.

Step 2: The curvature components in the two directions can be obtained from the intensity changes in the two directions. According to the results in Fig. 3d, the intensity variation exhibits a linear relationship with the curvature component, denoted as ***s***; the maximum value of ***s*** (i.e., ***s***_***m***_) corresponds to the bending direction along the line connecting the fiber axis and the scatterer, which is a key parameter for calculating the two-dimensional bending direction and curvature, and its value in the experiment is 0.05 dB·m^-1^.

1 $${\kappa }_{i}=\Delta {I}_{i}\cdot {s}_{m}$$

Step 3: Separate the curvature components along the X-axis and Y-axis: assign the curvatures at odd-numbered positions to the ***κ***_*xi*_, and those at even-numbered positions to the ***κ***_*yi*_.

Step 4: The total curvature and the bending angle with respect to one of the axes can be simply calculated from the curvature components of the orthogonal type distribution. Here we choose the reference axis as the X-axis, and the angle between the X-axis and the actual reference plane can be obtained by a simple calibration. The formulas for curvature calculation and angle calculation are:

2 $${\kappa }_{j}=\sqrt{{(({\kappa }_{xi})/2)}^{2}+{(({\kappa }_{yi})/2)}^{2}}$$

3 $${\theta }_{j}=\arctan ({\kappa }_{yi}/{\kappa }_{xi})$$

Among them, *i* takes the value range of 1-36, representing the scatterers; *j* takes the value range of 1-35, representing the sensing point.

Step 5: Interpolate the spline function on and to get and differentiate on to get the torsion function.

Step 6: Bring into the Frenet framework:

4 $$\begin{array}{c}T^{\prime} (j)=\kappa (j)N(j)\\ N^{\prime} (j)=-\kappa (j)T(j)+\tau (j)B(j)\\ B^{\prime} (j)=\tau (j)N(j)\end{array}$$

where *T(j)* is the tangent vector, *N(j)* is the normal vector and *B(j)* is the sub-normal vector, and the tangent vector function *T(j)* is obtained by numerical solution. The coordinates of each point in space will then be obtained by integrating over the arc length:

5 $$R(j)=\int T(j)dj+{R}_{0}$$

Step 7: Fit a spline function to the calculated discrete points to get a smooth curve in space.

### Error calculation method

In order to evaluate the accuracy of the curve reconstruction results, we used two judging criteria: relative error and end error.

The relative error, also known as the root-mean-square error, is mainly used to evaluate the overall reconstruction accuracy of the reconstructed curves compared with the standard curves, with smaller values indicating higher reconstruction accuracy. The calculation formula is:

6 $$\mathrm{Relative}\,\mathrm{error}=\sqrt{\frac{\displaystyle \sum _{i=1}^{35}{({x}_{i}-{x}_{r})}^{2}+{({y}_{i}-{y}_{r})}^{2}+{({z}_{i}-{z}_{r})}^{2}}{n}}$$

where *(x*_*i*_*, y*_*i*_*, z*_*i*_*)* denotes the coordinates of the reconstructed points and *(x*_*r*_*, y*_*r*_*, z*_*r*_*)* denotes the coordinates of the reference curve.

The second one is the end error, which is mainly used to evaluate the absolute error between the reconstructed curve and the end point of the reference curve, and the calculation scheme is:

7 $${\rm{End}\,{error}}=\sqrt{\frac{{({x}_{e}-{x}_{re})}^{2}+{({y}_{e}-{y}_{re})}^{2}+{({z}_{e}-{z}_{re})}^{2}}{L}}$$

where *(x*_*e*_*, y*_*e*_*, z*_*e*_*)* denotes the error at the end of the reconstructed curve and *(x*_*re*_*, y*_*re*_*, z*_*re*_*)* denotes the error at the end of the reference curve.

## Supplementary information

Supplementary information for Single-fiber three-dimensional shape sensing via femtosecond laser inscribed orthogonal eccentric scatterers

Preparation process of MOESs

## Data Availability

The data that support the findings of this study are available on the proper request from the corresponding authors.
