# Supplementary material for: Single-fiber three-dimensional shape sensing via femtosecond laser inscribed orthogonal eccentric scatterers
Source: Light Sci Appl. 2026 Aug 10;15:343. doi: 10.1038/s41377-026-02425-z (PMC13454164; doi:10.1038/s41377-026-02425-z)
Supplement: Supplementary file 1 [file 41377_2026_2425_MOESM1_ESM.docx]

**Supplementary information**

**Single-fiber three-dimensional shape sensing via femtosecond laser inscribed orthogonal eccentric scatterers**

*Pengtao* *Luo ^1^, Fengyi Chen ^1,*^, Tuan Guo^2^*, *Xueguang Qiao ^1^, Ruohui Wang ^1,*^*

*1. Xi'an Key Laboratory of Optical Fiber Sensing Technology for Underground Resources, School of Physics, Northwest University, Xi’an, 710127, China.*

*2. Institute of Photonics Technology, College of Physics & Optoelectronic Engineering, Jinan University, Guangzhou, Guangdong, 510632, China.*

*Email Address: chenfy@nwu.edu.cn (F.C.), rwang@nwu.edu.cn (R.W.)*

**Figure S1: The reel-to-reel femtosecond laser direct writing system. a The schematic components of the system. b Photos of the inscribing part of the system.**

**Figure S2: Comparison between multi-channel OFDR and MOESs fiber configuration.**

**Figure S3: Backscattering profiles of scatterers fabricated with different misplacement distances, clearly distinguishing the position of scatterers with millimeter-length misplace, with an offset of 4 μm.**

The spatial localization accuracy of the OFDR measurement was set to 100 µm, meaning the misplacement distance of the eccentric scatterers must exceed this value. Figure S3 shows the backscattering profiles of the eccentric scatterers array with misplacement distances of 2, 4, 5, 10, and 20 mm. Consequently, a misplacement distance of 2 mm for the eccentric scatterers array was sufficient to distinguish discrete scattering signals. The misplacement distance represents the spatial resolution of deformation encoding, which can reach the millimeter range.

**Figure S4: Relationship between scatterer offset and bending sensitivity. a Schematic illustration of eccentric scatterers inscribed at varying radial offsets from the core–cladding interface. b Measured bending sensitivity as a function of scatterer offset, showing an inverse Gaussian trend.**

The peak intensity of backscattered light from an eccentric scatterer is related to its offset from the fiber core. In a single-mode fiber, the optical field follows a Gaussian distribution, resulting in weaker back-reflected signals from scatterers located closer to the edge of the mode field. In contrast, when the fiber is bent, scatterers with larger offsets exhibit stronger variations in backscattered intensity—that is, higher bending sensitivity. This behavior arises mainly from two factors: (i) scatterers with greater offsets experience higher stress during bending, enhancing scattering due to deformation and the photoelastic effect; and (ii) the relative intensity change at the mode field edge is more pronounced under bending.

To investigate this, we fabricated scatterers with varying offsets and measured their bending sensitivity, as shown in Figure S4. Taking the core–cladding interface as the zero-offset position, scatterers were inscribed outward with 1 µm steps. The resulting sensitivity profile (Figure S4 b) follows an inverse Gaussian trend.

Given the inverse relationship between peak scattering intensity and bending sensitivity, an offset of 4 µm was chosen in this work to balance signal clarity with bending responsiveness. The corresponding backscattered signals from the fabricated scatterer array are shown in Figure 2g of the manuscript.

**Figure S5: Fluctuations in scattering intensity measured along the X and Y-axis.**

Although the signal peak intensity of each scatterer in Figure 3 exceeds 30 dB, the relatively low overall intensity (~-70 dB) poses challenges for signal measurement stability. To investigate the stability of intensity measurements, the sensing fiber with eccentric scatterers was fixed straight, and backscatter signals were recorded over 100 consecutive measurements. After peak detection and separation of X/Y-axis data, the standard deviation of intensity fluctuations was calculated, as shown in Figure S5. The minimum and maximum standard deviations of intensity fluctuations for scatterers along the X-axis were 0.1 dB and 0.12 dB, respectively, while for scatterers along the Y-axis, the range was between 0.09 dB and 0.13 dB. These intensity fluctuations lead to errors in curvature and angle calculations, thereby increasing the error in deformation encoding. To address this issue, random noise was reduced by averaging multiple measurements during signal processing, improving measurement accuracy to a certain extent.

**Figure S6: The angle, curvature, and torsion results of the circular arc curve with a bending radius of 6-12 cm.**

**Figure S7: Trend of end error variation under different sensing lengths**

**Figure S8: Variation in the backscattered signal profile with increasing fiber temperature.**

**Figure S9: Changes in the backscattered signal profile as axial strain increases.**

**Figure S10: Comparison of deformation encoding performance between MOESs array fiber and other methods.**

**Table S1: Comparison of key parameters of shape sensors**

|  | **Sensing fiber**  **(element)** | **Sensor points** | **Error** | **Size(µm)** | **Channel** | **Anti-interference** |
| --- | --- | --- | --- | --- | --- | --- |
| [20] | MCF(OFDR) | / | 2.5% | 125 | 4 | Yes |
| [30] | MCF(FBG) | 60 | 1.08% | 150 | 7 | No |
| [31] | MCF(FBG) | 20 | 4.51%-10.81% | 150 | 7 | No |
| [33] | Fiber bundle(FBG) | / | ＜1% | ＞1000 | 3 | No |
| [38] | SMF(CWG-FBG) | 5 | 6.84%-23.76% | 125 | 1 | No |
| [39] | SMF(CWG-FBG) | 3 | ~1% | 125 | 1 | No |
| **This work** | **SMF(MOESs)** | **＞400** | **<8%** | **125** | **1** | **Yes** |

**Figure S11. Torsion compensation scheme. (a) Workflow of deformation reconstruction with torsion compensation. (b) Encoding rule for torsion correction, showing the angle reconstruction differences of four adjacent scatterers with and without torsion. (c-d) Reconstructed results of a fixed shape when the fiber is twisted from 0 to 13 rad/m, without (c) and with (d) torsion correction. (e) Change in end error before and after torsion correction, reduced from 132.5% to 53.6% at 13 rad/m. (f) Change in relative error before and after torsion correction, reduced from 101.6% to 36.1% at 13 rad/m.**

In some applications, fiber torsion may occur. To avoid torsional interference, solutions such as helical multi-core fibers and loose-tube packaging have been proposed. Here, we propose a new torsion correction scheme for eccentric scatterers. By rereviewing the coding rules of MOESs, since the sensing point is selected between two adjacent orthogonal scatterers, the bending at this point can also be calculated using spaced scatterers. Taking two adjacent groups of MOESs (four scatterers) as an example: X_1_, Y_1_, X_2_, Y_2_, the two coding rules of X_2_-Y_1_ and X_1_-Y_2_ yield the bending information of the same sensing point (upper part of Figure S10b). Assuming that the bending state and torsion within this small segment are uniform, when torsion occurs, there will be a deviation in the bending direction calculated by these two coding rules, and this deviation represents the torsional information. Subsequently, the bending curvature is further corrected (lower part of Figure S10b), which enables anti-torsion performance.

Based on the deformation reconstruction process in Figure 4a, steps for correcting the angle and curvature have been added. The simplified process is shown in Figure S10a. For experimental verification, the test fiber was placed in the aforementioned arc-shaped glass tube to ensure the actual shape remained unchanged. One end of the fiber was fixed, while the other end was twisted by a rotating translation stage. Tests were conducted with torsion levels ranging from 0 to 13 rad/m. The fiber shape reconstructed using the process in Figure 4a is shown in Figure S10c; although the actual shape was fixed, significant errors appeared in the reconstructed curve due to torsion. After compensating the curve using the proposed torsion correction scheme, the results are presented in Figure S10d. A comparison of the errors between the two schemes is shown in Figure S10e and f. At the maximum torsion level, the relative error was reduced from 101.6% to 36.1%, and the end-point error was decreased from 132.5% to 53.6%. It can be observed that the torsion compensation scheme played a certain role; the reason why the torsional effect was not completely eliminated is that fluctuations in intensity measurement cause significant deviations in angle calculation.

**Figure S 12：Concept of on-axis reference scatterers under large bending conditions**

In practical scenarios, optical fibers may experience sharp bending with small radii, leading to significant bending-induced loss and consequently affecting the intensity of subsequent MOESs signals. To better understand and potentially distinguish such effects, we introduce the concept of on-axis reference scatterers as a structural extension of the proposed sensing scheme.

The on-axis reference scatterer refers to a scattering point located the center of the fiber core, whose intensity variation is governed by different physical mechanisms compared to the off-axis MOESs scatterers. As illustrated in Fig. S12, the orange and blue markings still represent the X-axis and Y-axis, respectively, while the on-axis reference scatterers are marked in green. Under gentle bending conditions (i.e., relatively large bending radius), the optical field distribution near the fiber core center remains nearly unchanged, while the off-axis region undergoes more pronounced effective refractive index variations. As a result, the intensity of MOESs scatterers is primarily sensitive to bending direction, whereas the on-axis reference scatterer remains relatively stable.

In contrast, under tight bending conditions (i.e., small bending radius), where the total internal reflection condition is strongly perturbed, bending-induced loss becomes dominant. In this regime, both MOESs and on-axis scatterers exhibit a significant decrease in intensity. Therefore, the on-axis reference scatterer cannot compensate for or eliminate the influence of localized loss; instead, it can serve as an indicator to distinguish between bending-dominated and loss-dominated regimes.

It should be noted that this concept is introduced here as a qualitative extension to illustrate a possible strategy for handling extreme bending conditions.
